# A multi-level hypoglycemia early alarm system based on sequence pattern mining

**DOI:** 10.1186/s12911-021-01389-x

**Published:** 2021-01-21

**Authors:** Xia Yu, Ning Ma, Tao Yang, Yawen Zhang, Qing Miao, Junjun Tao, Hongru Li, Yiming Li, Yehong Yang

**Affiliations:** 1 College of Information Science and Engineering, Northeastern University, Shenyang, 110819 China; 2 Department of Endocrinology and Metabolism, Huashan Hospital, Fudan University, Shanghai, 200040 China

**Keywords:** Hypoglycemia early alarm, Sequential pattern mining, Prefix span, Diabetes mellitus

## Abstract

**Background:**

Early alarm of hypoglycemia, detection of asymptomatic hypoglycemia, and effective control of blood glucose fluctuation make a great contribution to diabetic treatment. In this study, we designed a multi-level hypoglycemia early alarm system to mine potential information in Continuous Glucose Monitoring (CGM) time series and improve the overall alarm performance for different clinical situations.

**Methods:**

Through symbolizing the historical CGM records, the Prefix Span was adopted to obtain the early alarm/non-alarm frequent sequence libraries of hypoglycemia events. The longest common subsequence was used to remove the common frequent sequence for achieving the hypoglycemia early alarm in different clinical situations. Then, the frequent sequence pattern libraries with different risk thresholds were designed as the core module of the proposed multi-level hypoglycemia early alarm system.

**Results:**

The model was able to predict hypoglycemia events in the clinical dataset of level-I (sensitivity 85.90%, false-positive 23.86%, miss alarm rate 14.10%, average early alarm time 20.61 min), level-II (sensitivity 80.36%, false-positive 17.37%, miss alarm rate 19.63%, average early alarm time 27.66 min), and level-III (sensitivity 78.07%, false-positive 13.59%, miss alarm rate 21.93%, average early alarm time 33.80 min), respectively.

**Conclusions:**

The proposed approach could effectively predict hypoglycemia events based on different risk thresholds to meet different prevention and treatment requirements. Moreover, the experimental results confirm the practicality and prospects of the proposed early alarm system, which reflects further significance in personalized medicine for hypoglycemia prevention.

## Background

Diabetes is a chronic disease that progresses over decades and can lead to serious complications such as retinopathy, neuropathy, and diabetic kidney disease [1]. Hypoglycemia endangers the life safety of diabetic patients and is the major limiting factor in the glycemic management of type 1 and 2 diabetes. When hypoglycemia occurs, hormone action in the body will lead to reactive hyperglycemia (Somogyi effect). Hypoglycemia can also stimulate the cardiovascular system and trigger arrhythmias, myocardial infarction, stroke, etc. Long-term and repeated severe hypoglycemia attacks can result in irreversible damage to the central nervous system. Furthermore, long-term hypoglycemia may even cause shock and death [2]. Therefore, predictive prevention and control of hypoglycemia events, timely prediction, and intervention of asymptomatic hypoglycemia, has a vital significance for the treatment of diabetic patients [3, 4].

With the development of CGM technology, the temporal order information of glucose fluctuations can be obtained and used for glucose management. One of the applications is designing hypoglycemia early alarm systems, which is an effective way to predict hypoglycemia events for diabetic patients. Generally, triggering hypoglycemia early alerts timely could facilitate patients to take a certain preventive intervention to avoid serious hypoglycemia events. In 2009, Buckingham [5] used linear projection and statistical prediction alarm methods to predict hypoglycemia events and provided a timely response (shutting down the insulin pump for 90 min). In 2010, Dassau [6] proposed a voting algorithm using five prediction models, containing linear projection, Kalman filtering, hybrid infinite impulse response filter, statistical prediction, and numerical logical algorithm, which achieved the prediction of hypoglycemia 35 min in advance. In 2013, Bayrak et al. [7] used recursive autoregressive partial least squares (RARPLS) algorithm to model the CGM sensor data and predict the future glucose concentration for the hypoglycemia alarm system. In 2014, Wang et al. [8] added sleep and exercise information based on the work of Bayrak and reset the threshold for alarm hypoglycemia events. In 2018, Yang [9] applied an autoregressive integrated moving average (ARIMA) model to predict the future blood glucose, to take appropriate measures in advance to prevent hypoglycemia or hyperglycemia. In 2020, Vehí [10] designed a hybrid model with four machine learning algorithms to solve the safety problems of diabetes management, including grammar evolution, support vector machines, artificial neural network, and data mining. Wonju [11] proposed a machine learning model with four sets of unique data-driven features, including random forest, support vector machine, K-nearest neighbor, and logistic regression algorithm. In the above studies, glucose prediction models [12] are mostly built based on CGM records and used to obtain the predictions in the short-term ahead. Then, the predictions are compared with the hypoglycemia threshold to judge the occurrence of hypoglycemia and conduct early alarm. However, for the glucose prediction model, it is unavoidable that the prediction value generally lags behind the real value, and the accuracy greatly affects the early alarm performance of hypoglycemia.

Sequential pattern mining [13] refers to the data digging process of searching frequent subsequences as patterns from the sequence database. As an important research topic of data mining, it has been adopted widely containing applications in language processing, alarm management, event management, and other fields [14–16]. Zhang et al. [17] proposed a sequence pattern mining method for communication networks based on topology constraints to find meaningful alarm sequence patterns and accordingly ensured the stability and reliability of the communication network. Claude [18] applied situational sequence pattern extraction techniques to epidemiological and meteorological data to determine the most important climatic factors of dengue fever and investigated the correlation between the extracted patterns and the early alarm of dengue fever outbreak in French Guiana. Yan et al. [19] generated the sequence mode of tunnel traffic events by the Prefix Span algorithm, which reflected the sequence characteristics of the tunnel traffic events and helped to build the tunnel traffic event rule base. Niyazmand et al. [20] used the improved Prefix Span algorithm to excavate the sequence pattern of the alarm data of the natural gas processing factory and used the obtained frequent sequence pattern to locate the cause of alarm flooding. Yasmin et al. [21] improved the classification algorithm based on sequential pattern mining, which not only improved the classification speed and extensibility but also maintained the accuracy of classification, thus solving the problem that it is difficult to mine high-precision numerical data. Sequential pattern mining aims to provide a way to decompose sequences into segments and assign them a value of confidence and support threshold, or more precisely, frequent patterns for finding segments within a time window. Plenty of previous studies have demonstrated the effectiveness of sequential pattern mining and its applicability to alarm management and analysis.

Sequential pattern mining based on a time series database focuses on the relationship among single events within the same transaction and among transactions, which to some extent can avoid the impact of model prediction error on the accuracy of hypoglycemia early alarm. In this paper, a sequential pattern mining algorithm is applied to the early alarm of hypoglycemia. Through symbolizing the historical CGM records, pattern mining is used to complete the construction of the hypoglycemia pattern library, to provide sufficient glucose control time. By applying the sequential pattern mining technique to CGM records, hidden information could be discovered to help clinicians and patients make better decisions. Besides, a multi-level hypoglycemia early alarm mechanism with different risk levels is designed based on hypoglycemia sequential pattern mining. Under the framework, clinicians can formulate reasonable hypoglycemia defense guidelines through expert knowledge or clinical experience in different situations. Therefore, a well-designed multi-level hypoglycemia early alarm system plays an important role in improving comprehensive glucose management and in avoiding hypoglycemia events, which has great significance for clinical treatment.

Based on the above ideas, the main content of this paper is shown as follows: Section “Methods” introduces the method of correlation sequence pattern mining and the multi-level hypoglycemia early alarm mechanism; Section “Results” shows the test results and analyzes the system performance; Section “Discussion” makes a discussion; Section “Conclusions” finally gives the conclusion.

## Methods

### Prefix span

Prefix Span [22] is a kind of effective mining algorithm for sequential patterns based on an incremental sequential pattern, without producing candidate sequences. The basic idea is that all possible frequent subsequences are not considered when the database is projected. Only checking the prefix subsequence is needed, and then their corresponding suffix subsequences are projected into the projection database. In each projection database, sequential patterns are grown by exploring local frequent patterns, and the setting of the support threshold and the risk threshold is the key to the algorithm [23]. The Prefix Span, which overcomes the disadvantage of FreeSpan in the construction of the projection database, mines the sequential pattern seen as creating a tree. In terms of the mining time, Prefix Span has a significant advantage compared with other algorithms such as FreeSpan [24], Apriori [25], and GSP [26]. Therefore, the Prefix Span algorithm is selected as the sequential pattern mining algorithm in this paper, and its relevant definitions are listed as follows.

#### Definition 1

Subsequence. For sequence ${\text{A}} = < a_{1} ,a_{2} , \ldots ,a_{n} >$ and sequence ${\text{B}} = < b_{1} ,b_{2} , \ldots ,b_{n} >\left( {{\text{n}} \le {\text{m}}} \right)$, if there is a number sequence ${1} \le j_{1} \le j_{2} \ldots \le j_{n} \le {\text{m}}$ meeting $a_{1} \epsilon b_{j1} ,a_{2} \varepsilon b_{j2} , \ldots ,a_{n} \epsilon b_{jn}$, then A is regarded as a subsequence of B.

#### Definition 2

Support threshold. If there is a sequence database D, the support threshold is expressed as the ratio between the number of sequences containing sequence S in the sequence database D and the total number of sequences D, namely, sup.

#### Definition 3

Frequent sequences. Sequence A frequently occurs in the sequence set and the support threshold of A in the sequence set is more than or equal to the support threshold.

#### Definition 4

Prefix. Given sequence ${\text{A}} = < a_{1} ,a_{2} , \ldots ,a_{n} >$ and sequence ${\text{B}} = < b_{1} ,b_{2} , \ldots ,b_{n} > \left( {{\text{n}} \le {\text{m}}} \right)$, if $b_{i} = a_{i} , \left( {i \le m - 1} \right),b_{m} \in a_{m}$, and all items in $\left( {a_{m} - b_{m} } \right)$ follow behind $b_{m}$, it is said that B is the prefix of A.

#### Definition 5

Projection. For the given sequence A and B, if B is the subsequence of sequence A, the projection A′ of A corresponding to the prefix B needs to satisfy the requirement of making B as the prefix of A′, so A′ is the largest subsequence of A that meets the above conditions.

#### Definition 6

Suffix. Given sequences ${\text{A}} = < a_{1} ,a_{2} , \ldots ,a_{n} >$ and ${\text{B}} = < b_{1} ,b_{2} , \ldots ,b_{{m^{{\prime }} }} > \left( {{\text{n}} \le {\text{m}}} \right)$, sequence B is the subsequence of A. If $A^{{\prime }} = < b_{1} ,b_{2} , \ldots ,b_{p} > (m < p \le n)$ is the projection from sequence A onto subsequence B, the suffix of sequence A' onto subsequence B is $< b_{{m^{{\prime \prime }} }} ,b_{m + 1} , \ldots ,b_{p} >$ and $b_{{m^{{\prime \prime }} }} = (b_{m} - b_{{m^{{\prime }} }} )$.

#### Definition 7

Projection database. Suppose A is a sequence schema in sequence database S, and sequence B is prefixed by A, so the projection database of A is the suffix of all sequences prefixed by A in S relative to A, denoted as S|A.

### Longest common subsequence

The longest common subsequence algorithm [27] aims to obtain the longest sequence of two or more known sequences. Given two sequences X and Y, it means that sequence Z is a subsequence of X and Y when another sequence Z is both a subsequence of X and Y. For the given sequence X, Y, and Z, where sequence Z is the common subsequences of sequence X and Y, and the length of other common subsequences in sequence X and Y are all shorter than that of sequence Z. Then, sequence Z is defined as the longest common subsequence of sequence X and Y. The subsequence obtained at this time means that each element in the sequence belongs to the original sequence and inherits the order of the original sequence but is not necessarily continuous. For example, for the sequence X =  <ffghj> and Y =  <fghfgh> , the common sequence contains the sequential and continuous sequence <fgh> as well as the discontinuous sequence <ffgh>, so the longest common sequence of these two is <ffgh>. At present, the brute force method and dynamic programming algorithm are applied to solve the longest common subsequence problems [28, 29].

In this paper, dynamic programming is applied to solve the longest common subsequence problem according to the next four steps: state division, state identification, state transition, and boundary determination. By setting the two-dimensional array C[i, j], record the length of the longest common sequence that the sequence ${\text{X}} = < x_{1} ,x_{2} , \ldots ,x_{i} >$ is m and ${\text{Y}} = < y_{1} ,y_{2} , \ldots ,y_{j} >$ is n. Considering the recursive relation from the subproblem solution, the recursive equation can be obtained as follows:

1 $$C\left[ {i,j} \right] = \left\{ {\begin{array}{*{20}l} 0 \hfill & {i = 0\;or\;j = 0} \hfill \\ {C\left[ {i - 1,j - 1} \right] + 1} \hfill & {i,j > 0\;and\;x_{i} = y_{j} } \hfill \\ {\max \left( {C\left[ {i,j - 1} \right],C\left[ {i - 1,j} \right]} \right)} \hfill & {i,j > 0\;and\;x_{i} \ne y_{j} } \hfill \\ \end{array} } \right.$$

The solution of the longest common subsequence is reached by using the recursion method from the lower right corner of the constructed matrix. The method is as follows: Firstly, initialize the first row and column of the two-dimensional array to 0. If the two elements corresponding to the position of (i, j) in the array are equal, then the corresponding value of C[i, j] is C[i − 1, j − 1] + 1; If the two elements corresponding to the position (i, j) are different in the sequence, then the corresponding value of C[i, j] is the largest one in C[i, j − 1] and C[i − 1, j]. The values of all corresponding positions in the two-dimensional array are obtained recursively in turn. When the solution is completed for each element in the entire two-dimensional array, the length of the longest common subsequence is the last value in the lower right corner of the two-dimensional array. Finally, use the completed two-dimensional array matrix to backtrack all the longest common subsequence. The lower right corner of the matrix is the starting point, and all the routes are searched out by the exhaustive method.

### Hypoglycemia early alarm

#### Definition of hypoglycemia early alarm problem

Based on the Prefix Span, this paper reaches the mining of hypoglycemia early alarm sequence pattern and creates the hypoglycemia early alarm sequence library. The basic strategy for constructing the hypoglycemia model library can be listed as follows:

1. Obtain the existing CGM records.
2. The preprocessed CGM time series data is judged by the blood glucose in a fixed continuous period, and the hypoglycemia pattern library is built according to the result classification.
3. Remove frequent and redundant sequences from the database.

In this paper, the early alarm of hypoglycemia based on sequential pattern mining is divided into the construction of the hypoglycemia frequent sequence library and the pattern matching of the target blood glucose sequence. The related concepts related to pattern mining of the hypoglycemia early alarm sequence are defined in this paper [30].

##### Definition 1

**Fig. 1:**
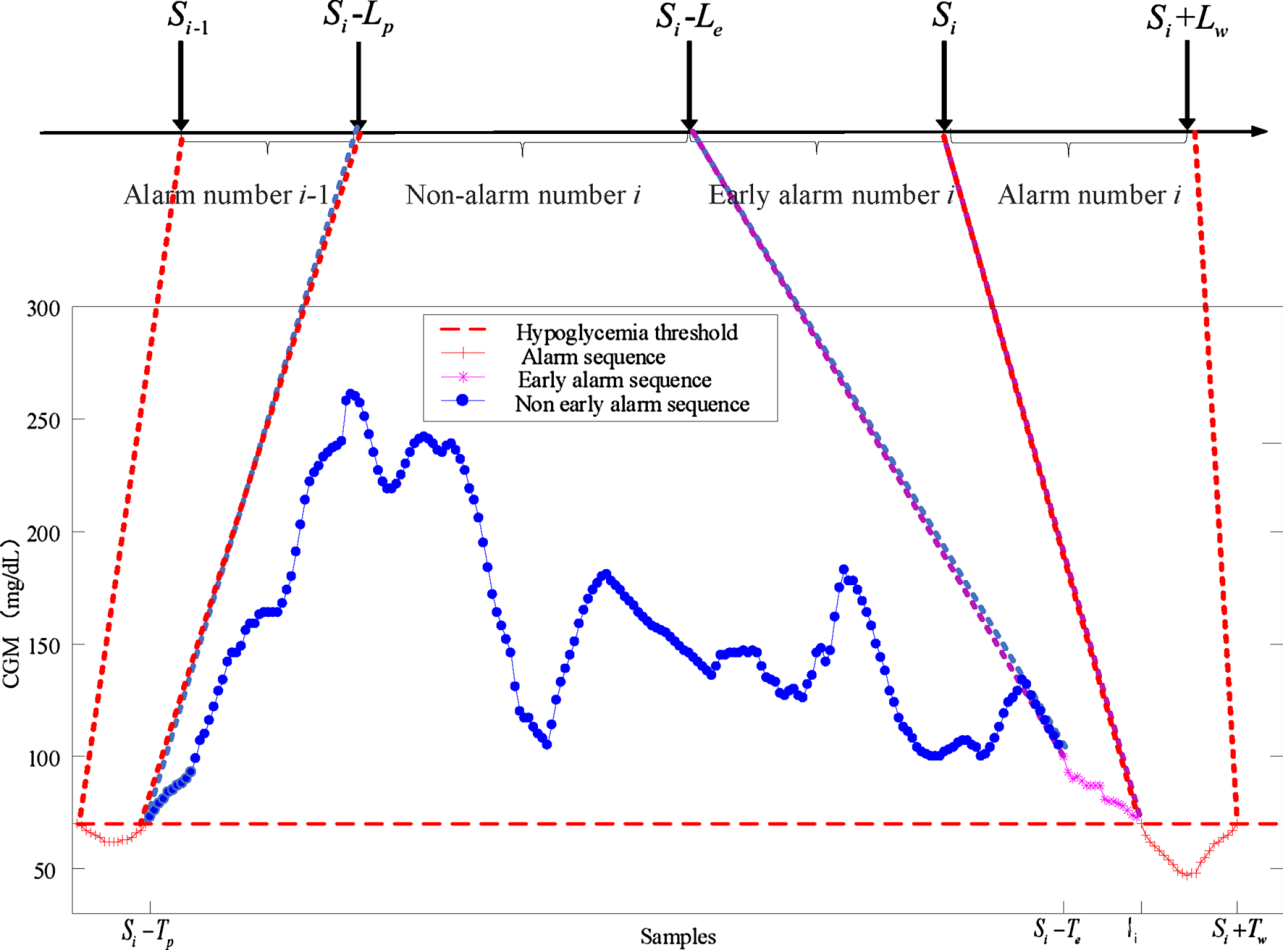
Hypoglycemia early alarm/non-alarm sequence

*An alarm event*. According to the alarm rule (blood glucose ≤ 3.9 mmol/L [31]), the starting index $S_{i}$ and the ending index $S_{i} + L_{w}$ of the alarm sequence is determined, as shown in Fig. 1.

##### Definition 2

*Early alarm sequence*. Given that the fixed window length is $L_{e}$, the early alarm sequence is composed of the glucose data that the number of it is $L_{e}$ before the alarm sequence. That is, the corresponding value in the sequence index ($S_{i} - L_{e}$) composes the early alarm sequence that the length is $L_{e}$.

##### Definition 3

*Non-alarm sequence*. Non-alarm sequence refers to the glucose sequence in the middle part of the alarm sequence and the next alarm sequence. That is, the sequence consisting of the corresponding values of the sequence index in (*S*_*i*_* − L*_*p*_) ~ (*S*_*i*_* − L*_*e*_). If the length of the non-alarm sequence is greater than that of the alarm sequence, the sequence needs to be processed in advance, which aims to ensure that the alarm and the non-alarm sequence are mined in the same sequence length.

#### Multi-level hypoglycemia early alarm

The pattern matching of the hypoglycemia sequence is based on the similarity of the sequence to predict the future glucose trend, which has a certain advantage over the prediction accuracy for early alarm. Between the construction of frequent sequential pattern libraries of hypoglycemia and the selection of hypoglycemia, a threshold exists a causal relationship. The framework process with multiple thresholds is reached by setting different hypoglycemia thresholds. Different thresholds can be given according to clinical needs or the actual needs of the patients. In this paper, hypoglycemia thresholds [32] (such as 3.0 mmol/L, 3.9 mmol/L and 4.4 mmol/L) are correspondence with the level-I, level-II and level-III libraries then refer to different early alerts respectively. The specific information were shown as follows:

- *Level-I (Red Alert with threshold 3.0 mmol/L):* represents the highest level of alert, which needs immediate measures to prevent adverse symptoms such as coma;
- *Level-II (Orange Alert threshold 3.9 mmol/L):* means to approach the diagnostic limit of hypoglycemia;
- *Level-III (Yellow Early Alert threshold 4.4 mmol/L):* means the occurrence of hypoglycemia, which suggests that attention should be paid to the blood glucose dynamics.

**Fig. 2:**
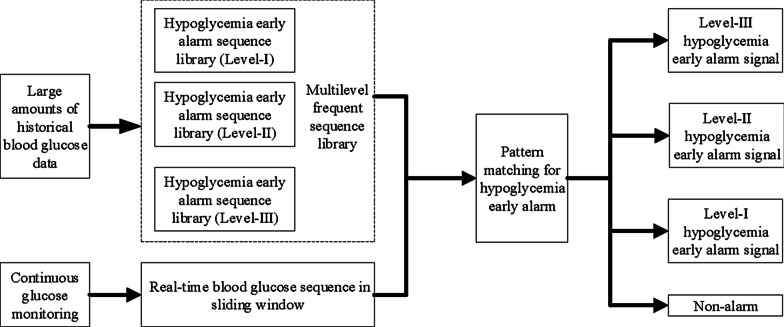
Pattern mining framework for multi-level hypoglycemia early alarm sequence

By matching the real-time glucose sequence with the frequent sequence libraries of the three early alarm levels, different matching results could be obtained to issue an early alert of different levels, as shown in Fig. 2. Different levels of early alarms enable diabetic patients to take appropriate treatment strategies according to their levels, which effectively avoids the early alarm time being too short to deal with. Therefore, a reasonable early alarm of hypoglycemia is an important means to effectively prevent and control adverse events.

#### The flow chart of multi-level hypoglycemia early alarm

**Fig. 3:**
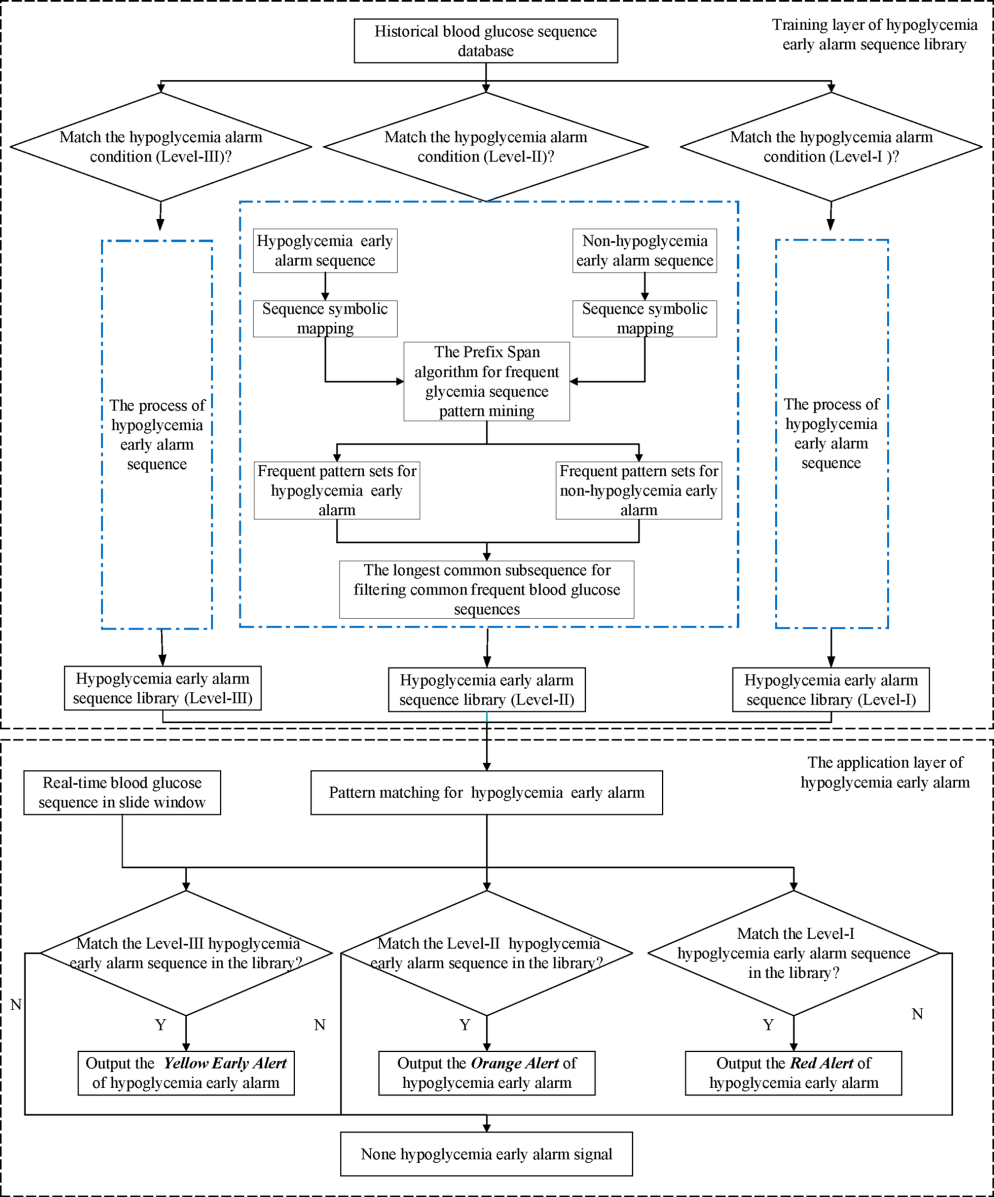
Multi-level pattern mining flow chart of hypoglycemia early alarm sequence

In this paper, the thought of sub-sequence pattern matching is used in hypoglycemia early alarm that the longest common subsequence is solved between the target sequence in the test set and the frequent subsequence in the early alarm pattern library. And the overall process is illustrated in Fig. 3. The main steps of the multi-level hypoglycemia early alarm are listed as follows:

- *Step 1:* In the original time series data, the alarm event data is screened out according to different levels of hypoglycemia early alarm rules, and the blood glucose data of patients is divided into an early alarm and non-alarm sequence set.
- *Step 2:* Symbolic mapping rules are used to symbolize the CGM records. On the one hand, it can ignore the high dimensional characteristic of time series; on the other hand, it can solve the problem that projection for each prefix may produce a large number of single items, which can improve the storage efficiency and speed up the processing.
- *Step 3:* The Prefix Span algorithm figures out the frequent glycemic sequence pattern mining of the early alarm and non-alarm sequence, which constructs the preliminary multi-level glucose sequence set.
- *Step 4:* The longest common subsequence algorithm is used to delete the frequent subsequences lower than the shortest length threshold in the alarm and non-alarm sequence library. The reason for this step is that the glucose sequence set formed in *Step 3* is a frequent sequence pattern set of all different levels meeting the support threshold, including frequent subsequences with the too long or too short sequence. The length of the shortest frequent subsequence $L_{min}$ is defined by the length of the early alarm sequence $L_{e}$ to reduce the probability of false positives.
- *Step 5:* Remove the common frequent sequence in the frequent and non-frequent alarm sequence in the frequent alarm pattern library by the longest common subsequence. And the longest common subsequence is applied to remove the redundant pattern in the current alarm sequence library and eventually complete the final establishment.
- *Step 6:* Multiple frequent sequence alarm pattern libraries with different levels are obtained in this step. Meanwhile, the real-time CGM records was slid with the same fixed window size as the alarm sequence to obtain the matching real-time sequence.
- *Step 7:* Match the divided real-time hypoglycemia sequence with multiple hypoglycemia early alarm pattern libraries of different levels by the longest common subsequence.
- *Step 8:* If the match is unsuccessful, the next sequence would be matched; If the match is successful, a corresponding level of hypoglycemia would be issued with three levels corresponding to yellow, orange, and red alert respectively.

## Results

### Dataset

In this paper, the clinical data from Huashan Hospital was used and it included the 2–5 days CGM records from 200 subjects in Type-2 Diabetes (T2D). The CGM records for a day consisted of 288 points measured every 5 min. Traditionally, diabetic patients with blood glucose concentration ≤ 3.9 mmol/L are diagnosed as hypoglycemia, which can be taken as the hypoglycemia threshold. This study was approved by the Ethics Committee of Huashan Hospital Affiliated to Fudan University, Shanghai, China. (No.2019-568).

As time series expressed in real numbers do not allow instant expression and extraction of patterns, most studies on sequential pattern mining are based on symbolic patterns. Therefore, this paper converted real data in a certain range into symbolic data, which was conducive to the generation of frequent patterns, as shown in Table 1. Measurements, which are close to the hypoglycemia thresholds, were divided at smaller intervals to fully present the glucose fluctuation.

**Table 1:** Blood glucose symbolic mapping rules for clinical data

Range of blood glucose	Symbolic representation	Range of blood glucose	Symbolic representation
(3.9,4.4]	‘a’	(10.5,11.8]	‘i’
(4.4,4.9]	‘b’	(11.8,13.3]	‘j’
(4.9,5.4]	‘c’	(13.3,14.8]	‘k’
(5.4,5.9]	‘d’	(14.8,16.3]	‘l’
(5.9,6.4]	‘e’	(16.3,22.2]	‘m’
(6.4,7.9]	‘f’	(3.0,3.9]	‘z’
(7.9,9.2]	‘g’	(2.2,3.0]	‘y’
(9.2,10.5]	‘h’		

### Evaluation indicators

In this experiment, indicators including sensitivity, false-positive, missing alarm rate, and early alarm were used to evaluate the performance of the hypoglycemia early alarm system based on sequential pattern mining. Metrics used to assess each of the methodologies using metrics of a positive example classified as true positive (TP), negative example classified as true negative (TN), negative example classified as false positive (FP), positive example classified as false negative (FN), the time of hypoglycemia defined as T_h_, and the time of the early alarm sequence endpoint defined as T_end_. And the formulas of evaluation indicators were as follows:

2 $${\text{Sensitivity}} = \frac{TP}{{TP + FN}}*100\%$$

3 $${\text{False - positive}} = \frac{FP}{{FP + TP}}*100\%$$

4 $${\text{Missing alarm rate}} = 1 - \frac{TP}{{TP + FN}}*100\%$$

5 $${\text{Early alarm time}} = {\text{T}}_{{\mathrm{h}}} - {\text{T}}_{{{\mathrm{end}}}}$$

### Results

To keep the frequent patterns obtained by sequential pattern mining at the same length, the fixed window was set at the same length as the early alarm event. There needed to be a support threshold for early alarm/non-alarm pattern set in the frequent pattern mining model. With the same support, the non-alarm mode produced a typical non-alarm sequence, excluding non-alarm information from the alarm sequence. Therefore, there were differences in the level of support for non-alarm and alarm sequence when dealing with the minimum support setting. The length of the shortest frequent subsequences is longer, the hypoglycemia sequence patterns obtained are less. Sequential pattern libraries are difficult to contain possible sequential patterns, resulting in an increased missing alarm rate. On the contrary, the sequence pattern explosively increases. A large number of false patterns appear, increasing the rate of false positives. We determined the length of the shortest frequent subsequence in the compromise between the missing alarm rate and the false alarm rate by several experiments.

First, clinical data was used to verify the feasibility of sequence pattern mining in hypoglycemia early alarm at a single level. The support and the sequence length of the system were set 0.2, 0.15, and 12 for all patients, and keep the length in the minimum alarm sequence model 1/2 of the alarm sequence length after cross-validation tests. The clinical data of 200 patients which contained 219 hypoglycemia, were selected for hypoglycemia sequential pattern matching. The hypoglycemia sequential pattern mining successfully forecasted 176 early alarms, 37 false alarms within an average early alarm time of 27.66 min. The sensitivity, false-positive, and missing alarm rate were 80.37%, 17.37%, and 19.63% respectively.

**Fig. 4:**
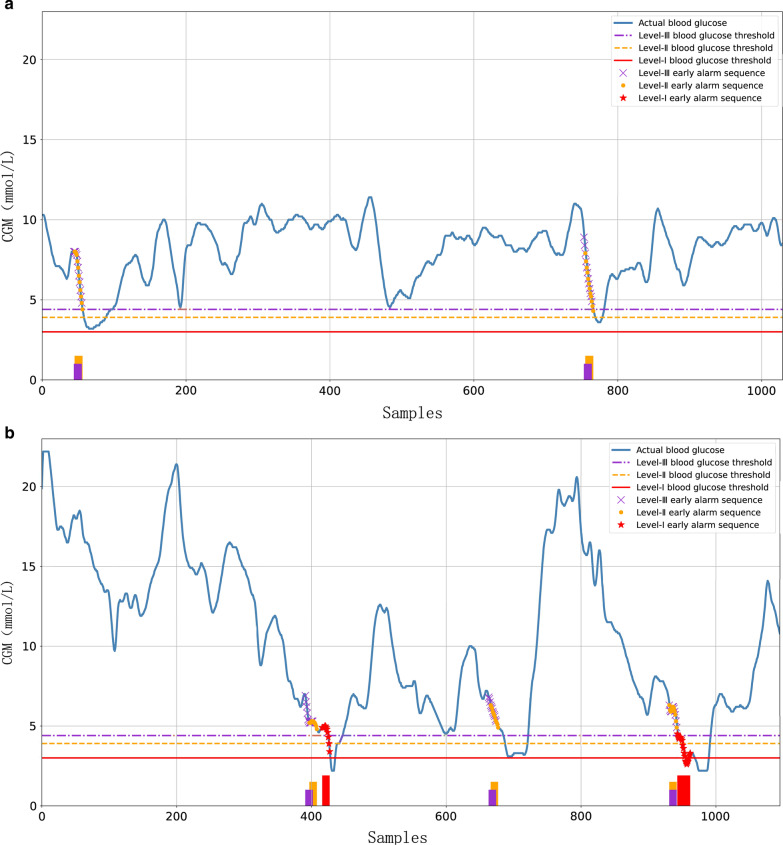
Effect of multi-level hypoglycemia early alarm

The multi-level early alarm system has a certain significance for patients and clinicians. We established a multi-level frequent sequence library for early alarm based on the feasibility of frequent sequence alarm for hypoglycemia. Firstly, the support and sequence length was set at the same level as previous clinical data settings. In the experiments, the hypoglycemia thresholds of clinical data were set at the level of 3.0 mmol/L, 3.9 mmol/L, and 4.4 mmol/L. The three levels were used to construct I, II, III frequent sequential patterns to support multi-level hypoglycemia early alarm. Then, the clinical data included 200 patients were applied to evaluate the multi-level system effect. The evaluation results were shown in Table 2, and the early alarm effects of two patients were shown in Fig. 4a, b.

**Table 2:** Multi-level early alarm effect result of 200 clinical patients

Alarm level	Actual hypoglycemia	Early alarm	False alarm	Average early alarm time	Sensitivity (%)	False-positive (%)	Missing alarm rate (%)
I	78	67	21	20.61	85.90	23.86	14.10
II	219	176	37	27.66	80.37	17.37	19.63
III	342	267	42	33.80	78.07	13.59	21.93

As shown in Fig. 4, three colors (purple, orange and red) refer to three levels of early alarm signals. level-III, II, I indicate general, worse, or severe hypoglycemia. It can be seen from Fig. 4a, b that multi-level hypoglycemia sequential pattern mining can provide some early alarms for T2D patients. The overall data in Fig. 4a shows dramatic fluctuations in the range of 3-12 mmol/L. The multi-level hypoglycemia early alarm system accurately provides timely early alarm for two-level hypoglycemia events of about 100 steps. Reasonable alarms were made for blood glucose fluctuations near 800 that exceeded the third and second blood glucose thresholds. It has a good early alarm effect for blood glucose fluctuations in a small range. When a patient receives a level-III early alarm, he or she needs to take measures to prevent hypoglycemia. And a reasonable level-III alarm can also reduce the possibility of severe hypoglycemia to a certain extent. For the patient in Fig. 4b, the blood glucose fluctuates sharply, and three severe hypoglycemia can be accurately early alerted. In comparison, the first level-III early alarm between 400–450 sampling step has a shorter lead time, but it can still trigger an alarm and provide enough time to take preventive measures. Data around the 600-sampling step, that fluctuates near the blood glucose threshold of level-III, can also be better distinguished, and the panic caused by false alarms to the patient can be avoided as much as possible.

## Discussion

According to the experiment results, the sequence pattern matching could predict impending hypoglycemia despite the presence of false alarms and missed alarms. With the early alarm information, patients can take measures in advance to avoid hypoglycemia events and improve the quality of glucose regulation. Missing alarms were probably caused by the unavailability of matching sequences with the necessary length as the first value of the patient's daily data was hypoglycemia. False alarms could be analyzed in following situations: Firstly, the symptoms of hypoglycemia contain great variation between individuals, and hypoglycemia thresholds should be set differently; Secondly, the patients are easy to actively replenish calories to avoiding hypoglycemia, especially after they feel hungry or take strenuous exercise.

In conclusion, frequent sequence pattern mining of hypoglycemia can improve alarm accuracy of possible hypoglycemia, which is applicable in alarm with sufficient time of preparation. In general, when level-III occurs, there may be a sustained drop or rise for glucose; level-II alarm probably appears following with level-III. Even though the level-III alarm is ignored, the alert prompt of the level-II would not be easily ignored. The multi-level early alarm of hypoglycemia has a certain application significance because the establishment of frequent hypoglycemia alarm libraries is based on a large amount of data, which greatly increases its accuracy. Most sequential pattern mining algorithms use a minimum support threshold to prune the combined search space. In this paper, the longest common subsequence is added to eliminate the non-alarm frequent sequences, which effectively distinguish the common frequent patterns of alarm and non-alarm sequences. It is feasible to mine the pattern of multi-level hypoglycemia sequence for early alarm. In the current researches, there are still several unavoidable problems of missing alarm since the current sequence pattern library cannot be updated in real-time. Besides, the initial hypoglycemia data volume is not large enough to match all the research targets. In a future study, the body characteristics of patients combined with the clinical experiences of doctors will be taken into more consideration and determine more appropriate parameters for patients in terms of more dynamic parameter selection and data-dependent characteristics.

## Conclusions

In this paper, the hypoglycemia prediction problem was transformed into the sequential state determination problem based on the sequential pattern mining method. We constructed a multi-level frequent hypoglycemia sequential pattern library through sequence pattern mining to realize a multi-level hypoglycemia alarm system with different thresholds, to meet different hypoglycemia risk prevention requirements. The clinical experimental results showed that the sequential pattern mining algorithm could identify the sequence characteristics and predict the future hypoglycemia events with relatively high precision. We believe that the proposed multi-level early alarm mechanism is helpful and flexible for doctors to set hypoglycemia thresholds with different risk levels according to various personalized clinical situations, and to make specific responses will reduce the occurrence of severe hypoglycemia events, and further avoid the risk of hypoglycemia and improve the quality of blood glucose management.

## Data Availability

The datasets used and/or analyzed during the current study are available from the corresponding author on reasonable request.

## Abbreviations

CGM: Continuous Glucose Monitoring
RARPLS: Recursive autoregressive partial least squares
ARIMA: Autoregressive integrated moving average
T2D: Type-2 diabetes
TP: True positive
TN: True negative
FP: False positive
FN: False negative
